# BRCA Status Dictates Wnt Responsiveness in Epithelial Ovarian Cancer

**DOI:** 10.1158/2767-9764.CRC-24-0111

**Published:** 2024-08-13

**Authors:** Hussein Chehade, Radhika Gogoi, Nicholas K. Adzibolosu, Sandra Galoforo, Rouba-Ali Fehmi, Mira Kheil, Alexandra Fox, Seongho Kim, Ramandeep Rattan, Zhanjun Hou, Robert T. Morris, Larry H. Matherly, Gil Mor, Ayesha B. Alvero

**Affiliations:** 1 Center for Molecular Medicine and Genetics, Wayne State University School of Medicine, Detroit, Michigan.; 2 Department of Obstetrics and Gynecology, C.S. Mott Center for Human Growth and Development, Wayne State University School of Medicine, Detroit, Michigan.; 3 Department of Pathology, Wayne State University School of Medicine, Detroit, Michigan.; 4 Karmanos Cancer Institute, Detroit, Michigan.; 5 Division of Gynecology Oncology, Department of Women’s Health Services, Henry Ford Cancer Institute and Henry Ford Health System, Detroit, Michigan.; 6 Department of Oncology, Wayne State University School of Medicine, Detroit, Michigan.

## Abstract

The association of *BRCA1* and *BRCA2* mutations with increased risk for developing epithelial ovarian cancer is well established. However, the observed clinical differences, particularly the improved therapy response and patient survival in *BRCA2*-mutant patients, are unexplained. Our objective is to identify molecular pathways that are differentially regulated upon the loss of *BRCA1* and *BRCA2* functions in ovarian cancer. Transcriptomic and pathway analyses comparing *BRCA1*-mutant, *BRCA2*-mutant, and homologous recombination wild-type ovarian tumors showed differential regulation of the Wnt/β-catenin pathway. Using Wnt3A-treated *BRCA1/2* wild-type, *BRCA1-*null, and *BRCA2-*null mouse ovarian cancer cells, we observed preferential activation of canonical Wnt/β-catenin signaling in *BRCA1/2* wild-type ovarian cancer cells, whereas noncanonical Wnt/β-catenin signaling was preferentially activated in the *BRCA1-*null ovarian cancer cells. Interestingly, *BRCA2-*null mouse ovarian cancer cells demonstrated a unique response to Wnt3A with the preferential upregulation of the Wnt signaling inhibitor Axin2. In addition, decreased phosphorylation and enhanced stability of β-catenin were observed in *BRCA2-*null mouse ovarian cancer cells, which correlated with increased inhibitory phosphorylation on GSK3β. These findings open venues for the translation of these molecular observations into modalities that can impact patient survival.

**Significance:**

We show that *BRCA1* and *BRCA2* mutation statuses differentially impact the regulation of the Wnt/β-catenin signaling pathway, a major effector of cancer initiation and progression. Our findings provide a better understanding of molecular mechanisms that promote the known differential clinical profile in these patient populations.

## Introduction

*BRCA1* and *BRCA2* are tumor suppressor genes with coding regions that are distinct from each other (1, 2). Both *BRCA1* and *BRCA2* function in homologous recombination (HR) to repair DNA double-strand breaks and maintain genome integrity (3, 4). Mutations in either protein confer an increased risk of breast, prostate, pancreatic, stomach, and ovarian cancers (4). There are more than 1,600 mutations identified in *BRCA1* and more than 1,800 mutations in *BRCA2* (5). These mutations lead to either frameshift, insertions, or deletions, resulting in missing or nonfunctional proteins. Mutations are either germline or somatic, with most somatic mutations being more common in disease state (6, 7).

Epithelial ovarian cancer causes the most deaths from gynecologic cancers. In the United States alone, more than 20,000 new cases are diagnosed, and more than 14,000 deaths are observed annually (8). Hereditary *BRCA1* or *BRCA2* germline mutations have been identified in 5% to 10% of epithelial ovarian cancers (9, 10), although tumor development in these individuals requires somatic inactivation of the remaining wild-type (WT) allele (11). Similar to sporadic tumors, BRCA-linked ovarian tumors mostly present as an advanced stage disease, with serous histology and moderate-to-high–grade disease (12). Interestingly, ∼5% to 7% of ovarian cancers are associated with somatic mutations in *BRCA1* or *BRCA2* (13), further highlighting the tumor suppressor function of these genes.

Despite their common roles in DNA repair, several clinical distinctions have been observed between *BRCA1* and *BRCA2* mutation carriers in the context of ovarian cancer. The lifetime risk for developing ovarian cancer is higher in carriers of *BRCA1* mutation (36% to 60%) than for *BRCA2* mutation carriers (16% to 27%; refs. 14, 15). The prevalence of ovarian cancer is also higher in *BRCA1* mutation carriers. Furthermore, 10% to 21% of women with *BRCA1* mutations will present with ovarian cancer by age 50 years compared with only 3% to 5% of women with *BRCA2* mutations (16, 17). Finally, although patients with *BRCA1/2* mutation survived longer when compared with patients with WT BRCA, direct comparison of *BRCA1* and *BRCA2* mutation carriers showed improved overall survival and improved response to chemotherapy in patients with *BRCA2* mutations (15, 18–22). The molecular mechanisms that confer this differential clinical profile between *BRCA1*- and *BRCA2*-mutant ovarian tumors are not well understood.

In this study, we sought to identify molecular pathways that are differentially regulated in the context of *BRCA1* and *BRCA2* mutations in ovarian cancer. Our results show that the loss of *BRCA1* versus *BRCA2* is associated with distinct responsiveness to Wnt signaling and β-catenin regulation. Our findings provide new insights into the molecular regulation of the Wnt pathway associated with *BRCA1* or *BRCA2* mutation.

## Materials and Methods

### Human subjects

The human samples used in our study were obtained with written informed consent in accordance with recognized ethical guidelines and approved by the Wayne State University Institutional Review Board (IRB-20-07-2521) and Karmanos Cancer Institute Institutional Review Board (IRB-20-13-052). Tumors were consecutively collected from patients diagnosed with high-grade serous ovarian cancer (HGSOC), fixed in 10% formalin for 72 hours, and embedded in paraffin. Patient demographics are shown in Table 1.

**Table 1:** Patient characteristics

Variable	All (*n* = 406)	*BRCA1* (*n* = 16)	*BRCA2* (*n* = 15)	Control (*n* = 375)	*P* value^a^
Age, years [median (range)]	61 (23, 87)	58 (35, 66)	61 (44, 81)	62 (23, 87)	0.217
Unknown	47	2	0	45	
Race, *n* (%)					0.895
White	253 (62)	9 (56)	10 (67)	234 (62)	
Black or African American	21 (5)	0 (0)	1 (7)	20 (5)	
Asian	4 (1)	0 (0)	0 (0)	4 (1)	
American Indian or Alaska Native	1 (0)	0 (0)	0 (0)	1 (0)	
Other race	3 (1)	0 (0)	0 (0)	3 (1)	
Unknown	124 (31)	7 (44)	4 (27)	113 (30)	
Stage, *n* (%)					0.814
Stage I	19 (5)	1 (6)	0 (0)	18 (5)	
Stage II	14 (3)	0 (0)	1 (7)	13 (3)	
Stage III	105 (26)	4 (25)	3 (20)	98 (26)	
Stage IV	111 (27)	5 (31)	6 (40)	100 (27)	
Unknown	157 (39)	6 (38)	5 (33)	146 (39)	

a Fisher exact test or Kruskal–Wallis rank as appropriate.

### Cell lines and culture conditions

Mouse ovarian cancer cell lines ID8^*Trp53−/−*^ (clone F3), ID8^*Trp53−/−*;*Brca1−/−*^ (clone 1.36), and ID8^*Trp53−/−*;*Brca2−/−*^ (clone 1.5) were kind gifts from Dr. Iain McNeish (Imperial College London, London, England; obtained in 2021). These isogenic cell lines were derived from WT ID8 mouse ovarian cancer cells (RRID: CVCL_IU14), and *Trp53*, *Brca1*, and *Brca2* were deleted using CRISPR/Cas9 (23, 24). Cells were cultured in DMEM high-glucose media (Thermo Fisher Scientific, Waltham, MA) supplemented with 4% FBS, 1% penicillin–streptomycin, 1% sodium pyruvate, and 1% insulin–transferrin–selenium. Cells were grown under typical culture conditions at 37°C with 5% CO_2_. All cell lines were frequently tested for *Mycoplasma* and authenticated at least once a year by short tandem repeat profiling and used within eight passages after thawing. Prior to Wnt3A treatments, cells were cultured in Opti-MEM I Reduced-Serum Medium (Thermo Fisher Scientific) overnight, followed by treatment with recombinant mouse Wnt3A in the same medium.

### Reagents

Recombinant mouse Wnt3A was purchased from R&D Systems. Cycloheximide was purchased from Tocris Bioscience.

### RNA sequencing and transcriptomic analysis

Ten 5-µm sections were obtained from formalin-fixed, paraffin-embedded (FFPE) blocks from each patient and used for RNA extraction. Only tumors with at least a 20% ratio of tumor nuclei to benign nuclei were included in the study. RNA extraction, cDNA library preparation, sequencing, and RNA sequencing (RNA-seq) data preprocessing were performed by Tempus as previously described (25). Transcriptomic data were used to determine the statuses of *BRCA1* and *BRCA2*. In our analysis, BRCA somatic mutations were defined as variants that result in loss of function of the BRCA protein at the cellular level, and HRwt samples were defined as those negative for aberrations in both *BRCA1* and *BRCA2*, as well as for 28 other HR genes (*ATM*, *ATR*, *ATRX*, *BARD1*, *BLM*, *BRIP1*, *CHEK1*, *CHEK2*, *FANCA*, *FANCC*, *FANCD2*, *FANCE*, *FANCF*, *FANCG*, *FANCI*, *FANCL*, *FANCM*, *MRE11A*, *NBN*, *PALB2*, *RAD50*, *RAD51*, *RAD51B*, *RAD51C*, *RAD51D*, *RAD52*, *RAD54L*, and *RPA1*).

Generated raw RNA-seq data were demultiplexed using BCL2FASTQ software v2.17 (RRID: SCR_015058). Quality control evaluation of the raw RNA-seq data was performed using MultiQC v1.11 (RRID: SCR_014982) with adapter sequences trimmed off using Skewer v0.2.2 (RRID: SCR_001151). Trimmed RNA-seq data were pseudoaligned to the Ensembl GRCh37 human reference and quantified using Kallisto v0.44 (RRID: SCR_016582). Gene-level abundance and corresponding transcripts per kilobase million values for 20,061 genes were provided for each sample by Tempus Labs and were used for our downstream analyses. Further bioinformatic analysis was performed in the R programming environment using RStudio v4.2.0 (RRID: SCR_000432). The edgeR package v3.38.4 (RRID: SCR_012802) was used to perform differential gene expression analysis. Genes with *P* values <0.05 and absolute log_2_ fold change (FC) >0.6 were considered differentially expressed. Volcano plots to visualize differential gene expression analysis results were generated using the ggplot2 package v3.4.0 (RRID: SCR_014601). Gene Ontology and Kyoto Encyclopedia of Genes and Genomes pathway impact analyses were performed using AdvaitaBio’s iPathwayGuide software. Significantly enriched Gene Ontology biological processes were defined as those with *P* values <0.05 using the smallest common denominator pruning method. Significantly impacted Kyoto Encyclopedia of Genes and Genomes pathways were defined as those with combined overrepresentation and pathway perturbation *P* values <0.05. Chord plots and dot plots for visualizing significantly impacted pathways or enriched biological processes were generated in R using the GOplot package v1.0.2 and ggplot2, respectively.

### RNA extraction, cDNA synthesis, and qPCR

Total RNA was extracted using the RNeasy Mini Kit (Qiagen) from cell pellets according to the manufacturer’s instructions. After DNase treatment, RNA was quantified, and purity was assessed using an Epoch microplate spectrophotometer (Agilent). One microgram of RNA was used to synthesize cDNA with the iScript cDNA Kit (Bio-Rad). A 1:10 dilution of cDNA was used for each qPCR reaction. qPCR was performed using SYBR Green Supermix (Bio-Rad) and run on the CFX96 PCR detection system (Bio-Rad) using the following thermocycling parameters: initial denaturation step for 2 minutes at 95°C; primer annealing step for 30 seconds at 55°C; elongation step for 60 seconds at 74°C for 30 cycles; and final extension step for 5 minutes at 74°C. Primers were synthesized by Integrated DNA Technologies, and sequences are shown in Supplementary Table S1. *PPIA* and β-actin were used as housekeeping genes. Relative expression was calculated using the comparative ΔΔCT method with ID8^*Trp53−/−*^ as reference. No RT samples were used as negative controls. All reactions were performed in triplicate.

### Protein lysis and cellular fractionation

Whole-cell protein lysates were isolated by resuspending cell pellets in 1× cell lysis buffer (Cell Signaling Technology) with added Complete Protease Inhibitor Cocktail (Millipore Sigma), followed by centrifugation for 20 minutes at 13,000 rpm. Cellular fractionation was performed using the NE-PER Nuclear and Cytoplasmic Fractionation Kit (Thermo Fisher Scientific) according to the manufacturer’s instructions. Protein lysates were quantified by bicinchoninic acid assay.

### SDS-PAGE and Western blot analysis

Fifty micrograms of protein lysate was electrophoresed on 12% SDS polyacrylamide gels and then transferred to polyvinylidene difluoride membranes (EMD Millipore). After blocking with 5% milk, the membranes were probed overnight with primary antibodies at 4°C and then incubated with an appropriate secondary antibody for 1 hour at room temperature. The blots were developed using enhanced chemiluminescence and imaged using a GE ImageQuant LAS 500 chemiluminescence imager (Cytiva Life Sciences).

The following antibodies were used: β-catenin (RRID: AB_823447), phospho-β-catenin (RRID: AB_331729), ILF3 (RRID: AB_10666431), Cox IV (RRID: AB_2085427), β-actin (RRID: AB_2923704), Tcf1/7 (RRID: AB_2199302), cyclin D1 (RRID:AB_2259616), c-Myc (RRID: AB_1903938), Axin2 (RRID: AB_10694569), phospho-GSK3β (RRID: AB_331405), and GSK3β (RRID: AB_2636978).

### Immunofluorescence

Cells were grown to ∼70% confluence in a four-well chamber, fixed with 4% paraformaldehyde, and permeabilized with 0.3% Triton X-100. Cells were then incubated with β-catenin antibody (RRID: AB_823447) or Phalloidin-iFluor 488 (Abcam) at 4°C overnight. Nuclei were stained with 4′,6′-diamidino-2-phenylindole (Thermo Fisher Scientific) prior to mounting with a glass coverslip. Cells were imaged using a Nikon Eclipse 90i fluorescent microscope with FITC and 4′,6′-diamidino-2-phenylindole channels at 20× magnification.

### IHC

IHC evaluation was performed on full sections. Anti–β-catenin (RRID: AB_294180) was used on formalin-fixed, paraffin-embedded tissue sections. Specimen preparation involved heating the deparaffinized tissue sections in a water bath (95–99°C) prior to IHC staining. Silanized slides were used for greater adherence of tissue sections to glass slides. The staining procedure involved diluting the antibody with M3539 at 1:200 in Dako Antibody diluent, and the Dako Negative Control Mouse IgG1 was used as the negative control reagent. Dako EnVision+ kits were used for visualization. For staining interpretation, the cellular staining pattern was membranous, with neoplastic cells displaying nuclear and diffuse cytoplasmic staining. Positive controls were as follows: (i) normal ovarian tissue taken as an internal control for β-catenin IHC and (ii) a histologically diagnosed section of ovarian serous carcinoma for nuclear positivity by β-catenin IHC. A negative control was achieved by omitting the primary antibody.

IHC slides were evaluated blindly by two independent pathologists. Expression of β-catenin in cells was compared between normal stromal and neoplastic tissues. The criterion for a positive reaction was a staining of the cytoplasm and the membrane, with the cutoff for a negative result being <5% of cells stained with β-catenin (26). The staining percentage, intensity, and site (membranous vs. cytoplasmic) were recorded. The scoring of positive β-catenin expression was performed according to Mauri and colleagues (27). In brief, the staining intensity was scored as follows: 0, no expression; 1+, weak expression; 2+, moderate expression; 3+, strong expression; and 4+, very strong expression. The final score was expressed as the IHC staining score (IHC score) obtained by multiplying the percentage of positive cells with the staining intensity.

### *In vivo* studies

All *in vivo* studies were approved by the Wayne State University Animal Care and Use Committee (IACUC 22-03-4474), and all mice were housed at the Wayne State University Division of Laboratory Animal Resources. A total of 5 × 10^5^ ID8^*Trp53−/−*^, ID8^*Trp53−/−*;*Brca1−/−*^, or ID8^*Trp53−/−*;*Brca2−/−*^ cells were injected intraperitoneally in C57BL/6 mice (The Jackson Laboratory; *n* = 6). Abdominal width was measured twice a week and provided surrogate for intraperitoneal tumor growth. Abdominal width was graphed using GraphPad Prism v9.3.1 (RRID: SCR_002798), and statistical significance was calculated using two-way ANOVA. Experiments were terminated when the abdominal width reached 3.4 cm. These values were used to determine overall survival. Survival was graphed and calculated using GraphPad Prism v9.3.1. All mice were included in the analysis. Investigators were aware of group allocation.

### Statistical analysis

Unpaired two-tailed Student *t* tests assuming Gaussian distribution or one-way ANOVA with the Dunnett multiple comparisons test were used for comparison between different groups. *P* values of 0.05 or less were considered statistically significant. Data were graphed, and statistical analyses were performed using GraphPad Prism v9.3.1 (RRID: SCR_002798).

### Data availability

All data presented are available upon request to the corresponding author.

## Results

### The Wnt signaling pathway is differentially regulated in *BRCA1*-mutant and *BRCA2*-mutant high-grade serous ovarian tumors

**Figure 1:**
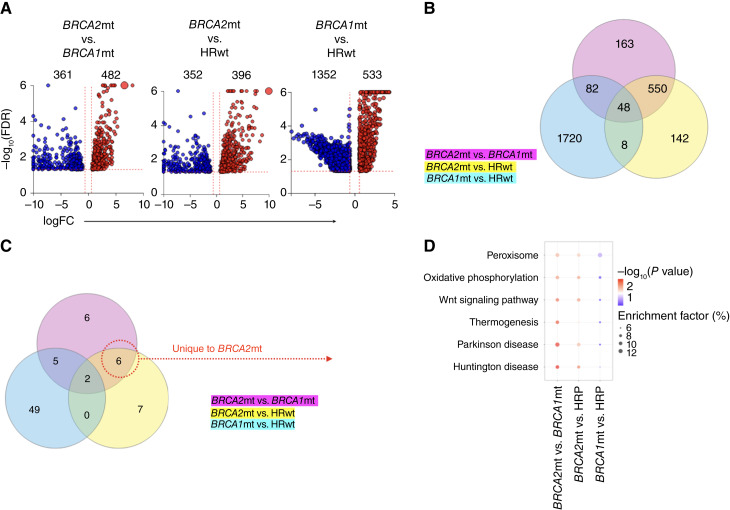
Transcriptomic differences between patients with *BRCA1*mt, *BRCA2*mt, and HRwt ovarian cancer. RNA-seq was performed on patients with HRwt (*n* = 375), *BRCA1*mt (*n* = 16), and *BRCA2*mt (*n* = 15) ovarian cancer. **A,** Volcano plots showing the distribution of DEGs (*P* = 0.05; FC > 1.5) in the compared groups as indicated. **B,** Venn diagram of DEGs in each of the groups compared showing 163 genes unique to *BRCA2*mt tumors. **C,** Venn diagram of differentially regulated pathways in each of the groups compared showing six pathways unique to *BRCA2*mt tumors compared with *BRCA1*mt and HRwt; these six pathways are shown in **D**.

To identify molecular pathways that are differentially regulated in the context of *BRCA1* or *BRCA2* mutation in ovarian cancer, we compared the transcriptomic profile of ovarian tumors obtained from patients with HR WT (HRwt; *n* = 375), *BRCA1*-mutant (*BRCA1*mt; *n* = 16), and *BRCA2*-mutant (*BRCA2*mt; *n* = 15) HGSOC (Table 1). After RNA-seq, we performed unpaired *t* tests to identify differentially expressed genes [DEG: *P* < 0.05 and FC >1.5] comparing *BRCA2*mt versus *BRCA1*mt, *BRCA2*mt versus HRwt, and *BRCA1*mt versus HRwt. From 18,284 genes with measured expression, 843 DEGs were found between *BRCA2*mt versus *BRCA1*mt, 748 DEGs between *BRCA2*mt versus HRwt, and 1,885 DEGs between *BRCA1*mt versus HRwt (Fig. 1A). Meta-analysis showed a substantial number of DEGs unique to each of these genetic subtypes, demonstrating distinct transcriptomic profiles between the compared groups (Fig. 1B).

To better understand the biological significance of these DEGs, we performed pathway enrichment analysis and identified 19 pathways that were differentially regulated in *BRCA2*mt versus *BRCA1*mt, 15 pathways that were differentially regulated in *BRCA2*mt versus HRwt, and 56 pathways that were differentially regulated in *BRCA1*mt versus HRwt. The top 10 differentially regulated pathways for each group comparison are shown in chord diagrams in Supplementary Figs. S1–S3. Following this, we conducted meta-analysis and using Venn diagrams, identified six pathways that were uniquely different in *BRCA2*mt tumors compared with *BRCA1*mt and HRwt tumors (Fig. 1C; overlap of pink and yellow circles). These pathways are peroxisome, oxidative phosphorylation, Wnt signaling, thermogenesis, Parkinson disease, and Huntington disease (Fig. 1D).

**Figure 2:**
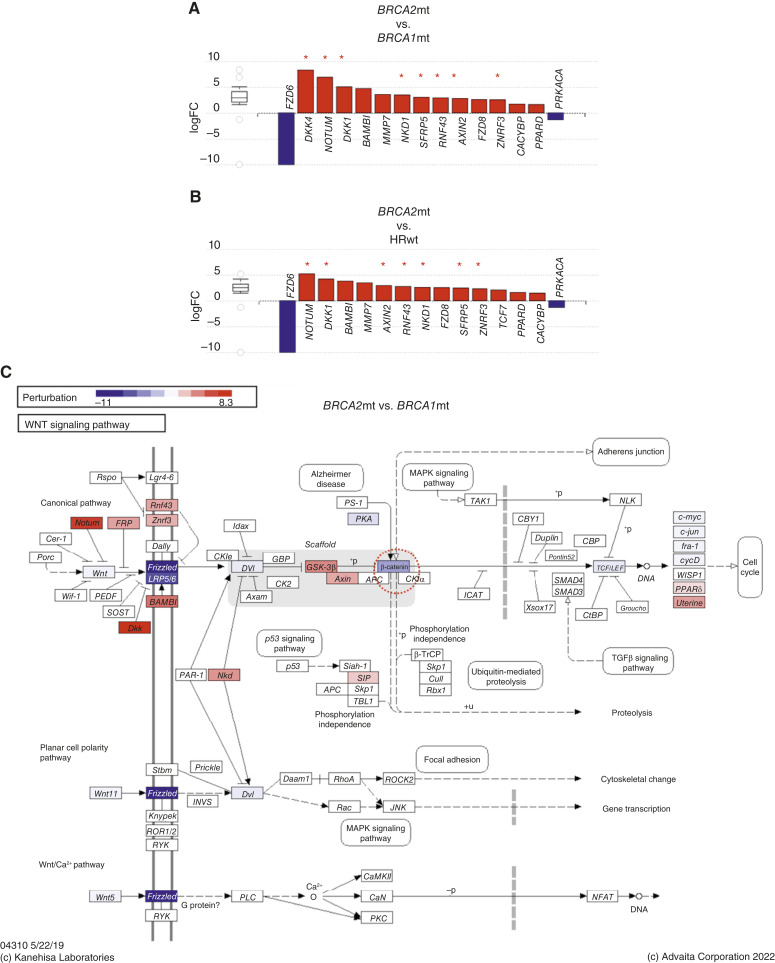
Wnt signaling is predicted to be inhibited in *BRCA2*mt ovarian tumors. **A,** DEGs comparing *BRCA2*mt vs. *BRCA1*mt and (**B**) *BRCA2*mt vs. HRwt reported as logFC; red bars represent upregulated, and blue bars represent downregulated; note that several Wnt inhibitors are upregulated in *BRCA2*mt tumors (red asterisk). **C,** Perturbation analysis of the Wnt signaling pathway comparing *BRCA2*mt and *BRCA1*mt tumors; red genes are predicted to be upregulated, and blue genes are predicted to be downregulated; the dashed circle shows β-catenin is predicted to be downregulated. Red arrows indicate upregulation of several Wnt signaling inhibitors at the level of the receptor.

The observed difference in the regulation of Wnt signaling is of particular interest given that the Wnt pathway has been shown to contribute to PARP inhibitor resistance (28). Interestingly, we found that the Wnt signaling pathway was uniquely differentially regulated in *BRCA2*mt tumors compared with *BRCA1*mt and HRwt tumors (Fig. 1C–E). Further analysis of DEGs in this pathway showed the upregulation in *BRCA2*mt tumors of genes known to inhibit Wnt signaling. When *BRCA2*mt ovarian tumors were compared with *BRCA1*mt tumors, eight genes (*NOTUM*, *SFRP5*, *RNF43*, *ZNRF3*, *DKK1*, *DKK4*, *NKD1*, and *AXIN2*) that negatively regulate Wnt signaling were upregulated in *BRCA2*mt tumors (Fig. 2A). These negative regulators of Wnt signaling were also upregulated in *BRCA2*mt tumors when compared with HRwt tumors (Fig. 2B). Not surprisingly, perturbation analysis predicted that the function of β-catenin is inhibited in *BRCA2*mt ovarian tumors compared with *BRCA1*mt and HRwt tumors (Fig. 2C; dashed circle). These results strongly suggest that *BRCA2*mt ovarian tumors are transcriptionally preprogrammed to respond differently to Wnt signaling compared with *BRCA1*mt and HRwt tumors.

### Inhibitors of Wnt signaling are overexpressed in *BRCA2-*null mouse cancer cells

To further characterize the potential correlation between BRCA status and responsiveness to Wnt signaling, we utilized the isogenic cells ID8^*Trp53−/−*^, ID8^*Trp53−/−*;*Brca1−/−*^, and ID8^*Trp53−/−*;*Brca2−/−*^. These cell lines were derived from the parental ID8 mouse ovarian cancer cell line and established using CRISPR/Cas9 to knock out *Trp53* and *Brca1* or *Brca2* (23, 24). The deletion of p53 makes these cells more representative of HGSOC (23, 24, 29–31). Using these cells, we measured the levels of *Dkk1*, *Axin2*, and *Notum* by qPCR and observed that the expression of all three genes was significantly higher in ID8^*Trp53−/−*;*Brca2−/−*^ compared with ID8^*Trp53−/−*^ and ID8^*Trp53−/−*;*Brca1−/−*^ cells (Supplementary Fig. S4A) and therefore correlated with the expression pattern observed in patient samples. The upregulation of Axin2 was validated at the protein level (Supplementary Fig. S4B).

To determine if the difference in these Wnt signaling genes can also be observed in other cancer types, we utilized a publicly available gene expression dataset for 4T1 *BRCA1*-null and 4T1 *BRCA2*-null mouse breast cancer cells (32). Wnt signaling was likewise differentially regulated when 4T1 *BRCA2*-null and 4T1 *BRCA1*-null cancer cells were compared (*P* = 0.013). Further analysis of DEGs in this pathway likewise showed upregulation of Wnt signaling inhibitors (Sfrp1, Notum, and Nkd2) in 4T1 *BRCA2*-null cells (Supplementary Fig. S5A). As such, perturbation analysis predicted that the Wnt signaling pathway is also inhibited in 4T1 *BRCA2-*null cells (Supplementary Fig. S5B). These results demonstrate that the effect of *BRCA2* loss on Wnt signaling is not limited to ovarian cancer cells and is found in other cancer types as well.

### *BRCA1-*null and *BRCA2-*null mouse ovarian cancer cells differentially respond to Wnt3A

**Figure 3:**
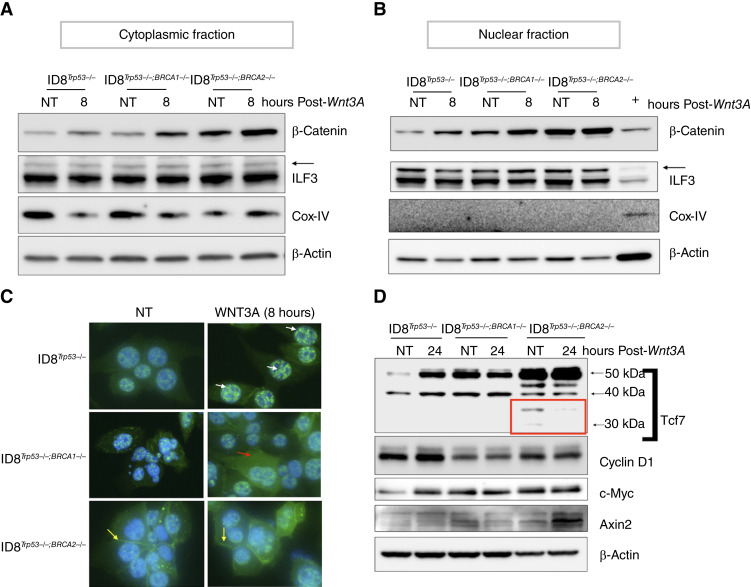
Characterization of the Wnt signaling pathway in isogenic mouse ovarian cancer cells upon loss of Brca1 or Brca2. Cellular fractionation was performed to separate cytoplasmic (**A**) and nuclear (**B**) fractions in control no-treatment cells or cells treated with 100 ng/mL Wnt3A for 8 hours; ILF3 and Cox-IV were used as integrity controls for nuclear and cytoplasmic fractions, respectively. **C,** Levels and cellular location of β-catenin were determined by immunofluorescence for β-catenin (green). DAPI nuclear staining is shown in blue. Images show both green and DAPI channels; white arrows show increased green staining in the nucleus after Wnt3A treatment in ID8^*Trp53−/−*^; the red arrow shows increased green staining in the cytoplasm after Wnt3A treatment in ID8^*Trp53−/−*;*Brca1−/−*^; yellow arrows show membranal staining in ID8^*Trp53−/−*;*Brca2−/−*^; images shown are specific foci in the cultures. **D,** Levels of canonical Wnt/β-catenin gene targets were determined by Western blot analysis using whole-cell lysates. The red box shows truncated forms of Tcf1/7. DAPI, 4′,6′-diamidino-2-phenylindole.

Our results so far suggest a differential regulation of the Wnt signaling pathway in BRCA2mt tumors. Indeed, of the 13 genes upregulated in BRCA2mt tumors, 5 are known to negatively regulate Wnt signaling at the level of the receptor LRP6 (Fig. 2C; red arrows). To determine if this molecular signature leads to a differential response, we treated ID8^*Trp53−/−*^, ID8^*Trp53−/−*;*Brca1−/−*^, and ID8^*Trp53−/−*;*Brca2−/−*^ cells with 100 ng/mL Wnt3A and characterized the propagation of Wnt signaling. This dose of Wnt3A has been used in multiple studies for *in vitro* treatment in both normal and cancer cells (33–35). First, we determined the effect of Wnt3A on β-catenin stabilization and cellular location by performing nuclear–cytoplasmic fractionation. In ID8^*Trp53−/−*^ and ID8^*Trp53−/−*;*Brca1−/−*^ cells, we observed the upregulation of β-catenin in both the nuclear and cytoplasmic fractions in response to Wnt3A (Fig. 3A and B). Interestingly, in ID8^*Trp53−/−*;*Brca2−/−*^ cells*,* the basal levels of β-catenin in both the cytoplasmic and nuclear fractions were already higher compared with basal levels in ID8^*Trp53−/−*^ and ID8^*Trp53−/−*;*Brca1−/−*^ cells. More importantly, ID8^*Trp53−/−*;*Brca2−/−*^ cells did not demonstrate a noteworthy upregulation in nuclear β-catenin in response to Wnt3A (Fig. 3B). We further confirmed these differences in β-catenin levels and location by immunofluorescence. Upon Wnt3A treatment, an increase in nuclear β-catenin staining was observed in foci of ID8^*Trp53−/−*^ cells as demonstrated by a more intense green staining in the nucleus in Wnt3A-treated cultures (Fig. 3C; white arrows). Quantification of nuclear β-catenin levels in all cells, however, did not show statistical significance between control and treated cultures, suggesting variability in response among cells in the culture (Supplementary Fig. S6). In foci of ID8^*Trp53−/−*;*Brca1−/−*^ cells, the Wnt-induced an increase in green fluorescence intensity which was mostly observed in the cytoplasm (Fig. 3C; red arrows). Similarly, however, quantification of cytoplasmic β-catenin levels did not show statistical significance (Supplementary Fig. S6). In ID8^*Trp53−/−*;*Brca2−/−*^ cells, Wnt3A treatment did not increase immunofluorescence for β-catenin compared with no-treatment control (Fig. 3C). Interestingly, similar to that observed with the cellular fractionation in Fig. 3A and B, immunofluorescence staining also showed that ID8^*Trp53−/−*;*Brca2−/−*^ cells have the highest basal levels of β-catenin compared with ID8^*Trp53−/−*^ and ID8^*Trp53−/−*;*Brca1−/−*^ cells (Fig. 3C). Furthermore, immunofluorescence showed membrane staining for β-catenin only in ID8^*Trp53−/−*;*Brca2−/−*^ cells (Fig. 3C; yellow arrows).

Finally, we determined the effect on known canonical Wnt/β-catenin gene targets, Tcf1/7, cyclin D1, c-Myc, and Axin2 (36). In ID8^*Trp53−/−*^ cells, we observed upregulation of full-length (FL) Tcf1/7 and c-Myc and to a lesser extent, cyclin D1 (Fig. 3D). Axin2 was not upregulated in ID8^*Trp53−/−*^ cells in response to Wnt3A*.* FL Tcf1/7, c-Myc, cyclin D1, and Axin2 were not upregulated in ID8^*Trp53−/−*;*Brca1−/−*^ cells. In ID8 ^*Trp53−/−*;*Brca2−/−*^ cells, Axin2, an integral component of the β-catenin destruction complex that confers an inhibitory effect on Wnt/β-catenin signaling (37), was the only target upregulated, and no changes were observed on FL Tcf1/7, c-Myc, and cyclin D1 (Fig. 3D). Interestingly, of the isogenic ID8 cell lines, basal levels of Tcf1/7 were the highest in ID8^*Trp53−/−*;*Brca2−/−*^ cells (Fig. 3D). Furthermore, only in ID8^*Trp53−/-*;*Brca2-/-*^ cells, we detected the appearance of truncated Tcf1/7 proteins (∼30 kDa; Fig. 3D; red box), which have been shown previously to have dominant-negative functions (38, 39). Taken together, these results demonstrate that changes in the status of *BRCA1* and *BRCA2* considerably affect the status Wnt/β-catenin pathway and the response to Wnt signaling.

### Loss of *BRCA2* is associated with β-catenin stability

**Figure 4:**
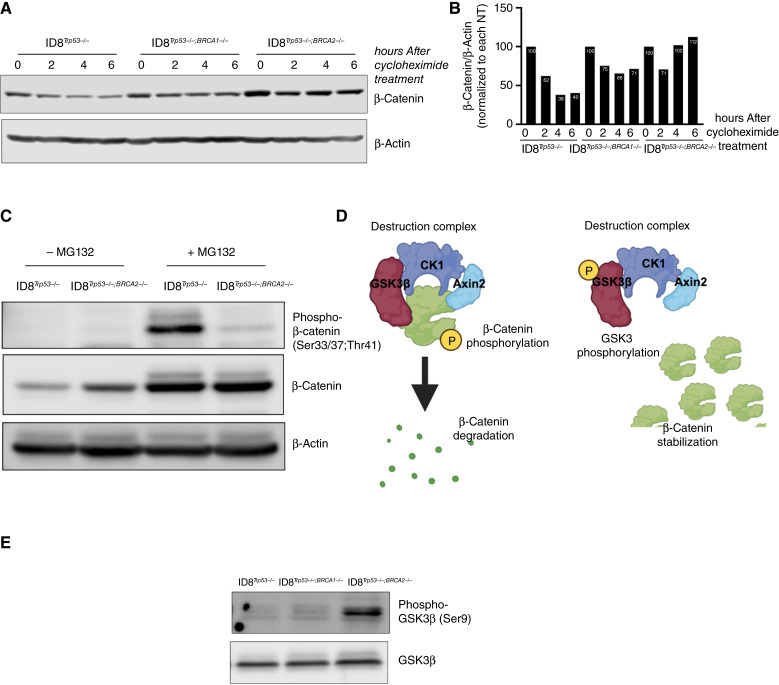
β-Catenin is stabilized upon loss of *BRCA2*. **A,** ID8 mouse ovarian cancer cells were treated with 10 µg/mL cycloheximide at designated time points, and levels of β-catenin were determined by Western blot analysis. **B,** Quantification of **A**. **C,** ID8 mouse ovarian cancer cells were treated with 50 µmol/L MG132, and levels of phosphorylated and total β-catenin were determined by Western blot analysis. **D,** Figures depicting components of the β-catenin destruction complex. **E,** Basal levels of phosphorylated and total GSK3β were determined by Western blot analysis. NT, no treatment. (Created with Biorender.com.)

Our next objective was to determine the molecular mechanisms that contribute to the high basal levels of β-catenin in the ID8^*Trp53−/−*;*Brca2−/−*^ cells. qPCR analysis showed no significant difference in the levels of *Wnt3A* or *Ctnnb1* mRNA between the three isogenic ID8 cell lines (Supplementary Fig. S7), demonstrating that the increased basal levels of β-catenin observed in ID8^*Trp53−/−*;*Brca2−/−*^ cells are not due to higher transcriptome levels of Wnt3A ligand nor β-catenin itself. However, treatment with cycloheximide to prevent new protein translation showed differential kinetics of β-catenin protein degradation. In ID8^*Trp53−/−*^ cells (Fig. 4A and B), we observed a steady decrease in the levels of β-catenin with ∼40% of the β-catenin pool remaining after 6 hours of cycloheximide treatment. In ID8^*Trp53−/−*;*Brca1−/−*^ cells, we also observed a modest decrease in the levels of β-catenin with ∼70% of β-catenin pool remaining after 6 hours of treatment (Fig. 4A and B). β-Catenin was most stable in ID8^*Trp53−/−*;*Brca2−/−*^ cells with >100% of β-catenin pool left post cycloheximide (Fig. 4A and B). These results suggest that the increased basal β-catenin expression observed in ID8^*Trp53−/−*;*Brca2−/−*^ cells reflect differences in stability, possibly due to alterations in the activity of the β-catenin destruction complex, which is the main regulator of β-catenin cellular levels.

The β-catenin destruction complex consists of GSK3β, casein kinase 1, adenomatous polyposis coli protein, Axin, and the ligase β-TrCP (40). The phosphorylation of β-catenin by GSK3β at residues Thr41, Ser33, and Ser37 targets β-catenin for subsequent ubiquitination by β-TrCP and proteasomal degradation (41, 42). We hypothesized that β-catenin is more stable in ID8^*Trp53−/−*;*Brca2−/−*^ cells because of perturbations in its phosphorylation status. To test this hypothesis, we determined the levels of phosphorylated β-catenin, specifically at the Thr41, Ser33, and Ser37 sites in ID8^*Trp53−/−*;*Brca2−/−*^ cells, and utilized ID8^*Trp53−/−*^ as a comparison. Given that β-catenin is constitutively degraded in ID8^*Trp53−/−*^, we first treated the cells with the proteasome inhibitor MG132 to prevent β-catenin degradation and therefore allow the interrogation of its phosphorylation status. As shown in Fig. 4C, MG132 treatment yielded comparable levels of total β-catenin in ID8^*Trp53−/−*^ and ID8^*Trp53−/−*;*Brca2−/−*^ cells. More importantly, our results showed that indeed β-catenin is phosphorylated in ID8^*Trp53−/−*^, but this phosphorylation is scarcely detected in ID8^*Trp53−/−*;*Brca2−/−*^ cells (Fig. 4C), suggesting the differential activity of GSK-3β in these cells.

The activity of GSK3β is also controlled by its phosphorylation (43). Phosphorylation of GSK3β (p-GSK3β) on Ser9 is associated with an inhibition of its kinase function (44) within the β-catenin destruction complex and can lead to the accumulation of β-catenin (Fig. 4D). We therefore investigated the levels of p-GSK3β. As shown in Fig. 4E and in line with our hypothesis, p-GSK3β was only observed in ID8^*Trp53−/−*;*Brca2−/−*^ cells. Taken together, our results show that in ID8^*Trp53−/−*;*Brca2−/−*^ cells, phosphorylation of GSK-3β (resulting in its inhibition) correlates with the accumulation (and stabilization) of unphosphorylated β-catenin.

### Loss of *BRCA1* is associated with the activation of noncanonical Wnt signaling

**Figure 5:**
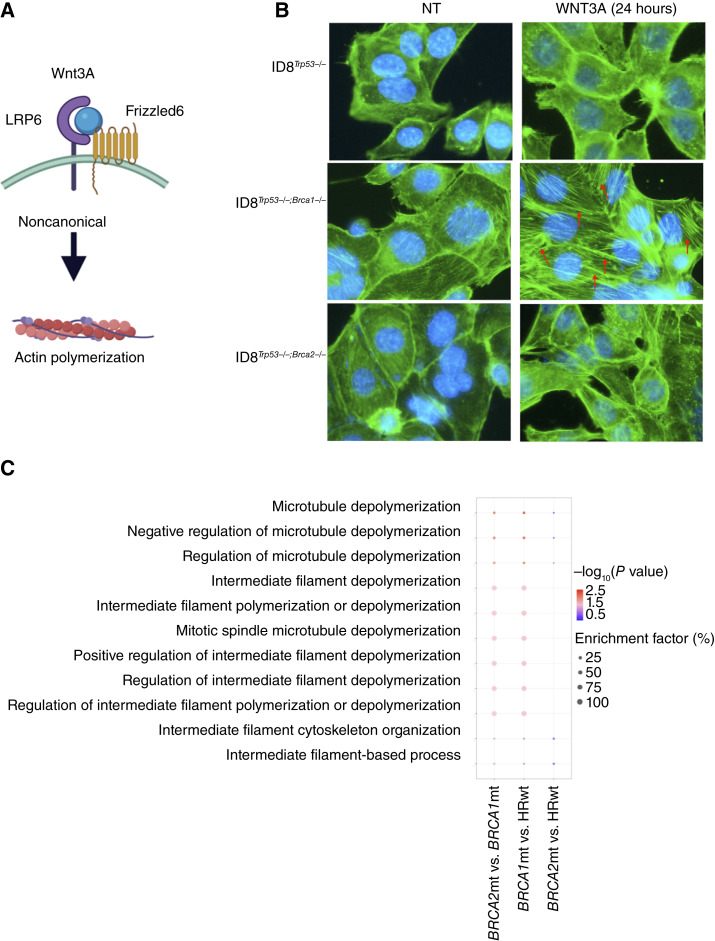
The noncanonical Wnt pathway is activated upon loss of *BRCA1*. **A,** Model of the noncanonical Wnt signaling pathway. **B,** ID8 mouse ovarian cancer cells were treated with 100 ng/mL Wnt3A followed by immunofluorescence for F-actin (green). DAPI staining is shown in blue. Red arrows point to F-actin filaments. Images show both green and DAPI channels. **C,** Biological processes related to filament and microtubule polymerization are differentially regulated in *BRCA1*mt vs. *BRCA2*mt and HRwt ovarian tumors. DAPI, 4′,6′-diamidino-2-phenylindole.

Having characterized the outcome of *BRCA2* loss on the Wnt/β-catenin signaling pathway, we then focused our attention on the outcome upon loss of *BRCA1* function. Our results so far demonstrated that in ID8^*Trp53−/−*;*Brca1−/−*^ cells, treatment with Wnt3A stabilized β-catenin mostly in the cytoplasm (Fig. 3A), but the transcription of canonical Wnt/β-catenin target genes was not observed (Fig. 3D). A closer look at the DEGs between *BRCA*1mt and *BRCA2*mt patient tumors showed the downregulation of *FZD6* in *BRCA*2mt compared with *BRCA1*mt (Fig. 2A). Another way to interpret this is that *FZD6* is upregulated in *BRCA1*mt tumors. FZD6 binds to Wnt ligands and propagates Wnt signaling preferentially toward the noncanonical pathway (Fig. 5A; ref. 45). The observation that *FZD6* is upregulated in *BRCA1*mt tumors provide a rationale to hypothesize that *BRCA1*mt tumors may respond to Wnt3A by activating the noncanonical pathway. We then investigated this pathway with focus on the planar cell polarity pathway by determining levels of filamentous actin (F-actin) by immunofluorescence. Our results show more filamentous staining for F-actin in ID8^*Trp53−/−*;*Brca1−/−*^ cells compared with ID8^*Trp53−/−*^ and ID8^*Trp53−/−*;*Brca2−/−*^ cells upon treatment with Wnt3A (Fig. 5B; red arrows). We then analyzed the patient transcriptomic data to determine if *BRCA1*mt tumors differentially regulate filament polymerization compared with *BRCA2*mt and HRwt tumors. Analysis of differentially regulated biological processes showed that processes involved in cytoskeletal organization and filament polymerization are indeed differentially regulated in *BRCA1*mt tumors compared with *BRCA2*mt and HRwt tumors (Fig. 5C). These processes are not differentially regulated in *BRCA2*mt compared with HRwt tumors (Fig. 5C). These results demonstrate that indeed the loss of *BRCA*1 preferentially directs Wnt signaling toward the noncanonical pathway affecting the cellular cytoskeleton.

### Decreased tumor growth and improved survival in ID8^*Trp53−/−*;*Brca2−/−*^-bearing mice

**Figure 6:**
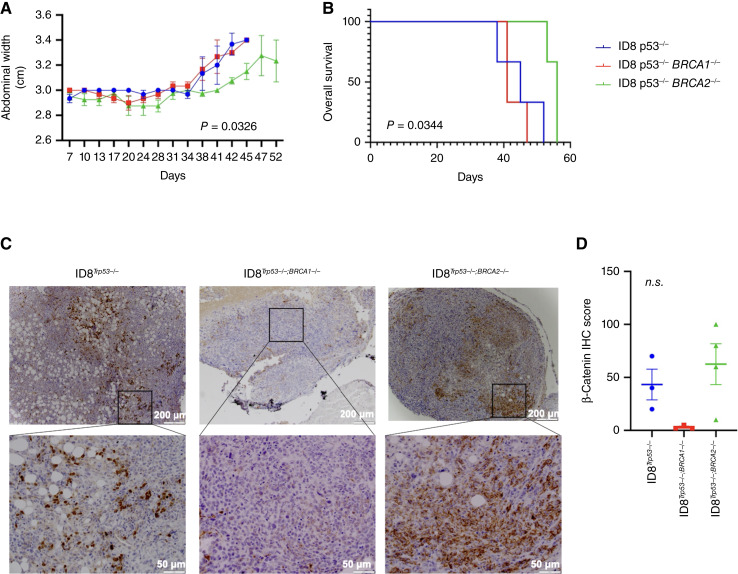
Decreased tumor growth kinetics and improved survival in mice bearing ID8^*Trp53−/−*;*Brca2−/−*^ cells. ID8 mouse ovarian cancer cells were injected intraperitoneally in C57BL/6 mice (*n* = 6). **A,** Abdominal width was used as surrogate for intraperitoneal tumor growth; note the significant decrease in tumor growth in mice bearing ID8^*Trp53−/−*;*Brca2−/−*^ cells. Data are presented as mean ± SEM. Two-way ANOVA with mixed-effect analysis was used to calculate statistical significance. **B,** Kaplan–Meier survival curve showing improved overall survival (defined as the day abdominal width reached 3.4 cm) in mice bearing ID8^*Trp53−/−*;*Brca2−/−*^ cells. The log-rank test was used to calculate statistical significance. **C,** Formalin-fixed, paraffin-embedded sections of intraperitoneal tumors were immunostained for β-catenin. **D,** Quantification of **C** as detailed in “Materials and Methods” (*n* = 3); ordinary one-way ANOVA with multiple comparisons was used to calculate statistical significance. *n.s.*, not significant.

Finally, we determined the effect of *BRCA1* or *BRCA2* loss on *in vivo* tumor growth. Thus, we injected the three ID8 isogenic cell lines intraperitoneally in C57BL/6 mice and monitored tumor progression by measuring abdominal width as a readout for intraperitoneal tumor burden. We observed significantly slower tumor growth kinetics in ID8^*Trp53−/−*;*Brca2−/−*^-bearing mice compared with ID8^*Trp53−/−*^*-* and ID8^*Trp53−/−*;*Brca1−/−*^-bearing mice (Fig. 6A). Overall survival was also significantly longer in ID8^*Trp53−/−*;*Brca2−/−*^-bearing mice with median survival of 45, 41, and 56 days for mice bearing ID8^*Trp53−/−*^, ID8^*Trp53−/−*;*Brca1−/−*^, and ID8^*Trp53−/−*;*Brca2−/−*^ cells, respectively (Fig. 6B). These findings recapitulate previous clinical observations of better prognosis in patients with *BRCA2*mt ovarian tumors (15).

To determine if the differential status of β-catenin is maintained *in vivo*, we determined its expression in the mouse tumors by IHC. Tumors from ID8^*Trp53−/−*;*Brca2−/−*^ cells demonstrated mostly membranal staining (Fig. 6C) and a higher β-catenin IHC score compared with tumors from ID8^*Trp53−/−*;*Brca1−/−*^ cells (Fig. 6D). Tumors from ID8^*Trp53−/−*^ cells showed mostly nuclear staining (Fig. 6C). These findings further demonstrated that β-catenin levels in the ovarian cancer cells and ovarian tumor microenvironment are regulated by BRCA status.

## Discussion

**Figure 7:**
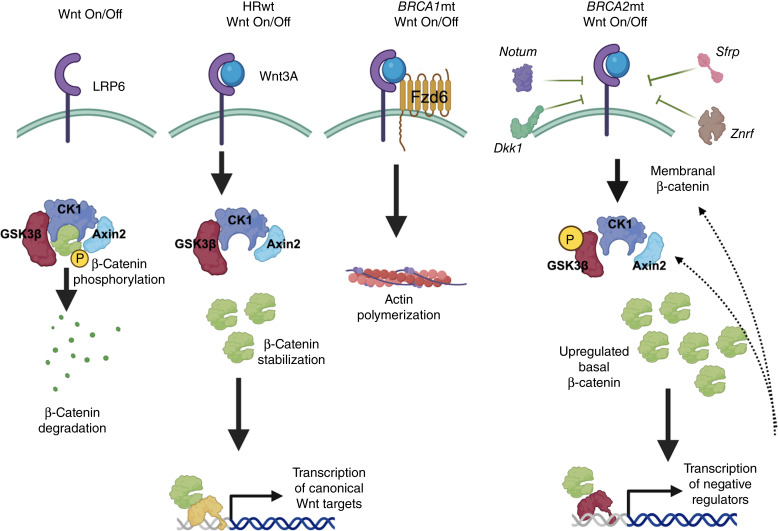
Proposed model showing differential response to Wnt3A in ovarian cancer cells in the context of BRCA mutation. Wnt3A activates the canonical Wnt/β-catenin pathway in ovarian cancer cells with WT *BRCA1* and *BRCA2*. Loss of *BRCA1* function leads to preferential activation of the noncanonical signaling pathway, leading to actin polymerization. Loss of *BRCA2* stabilizes β-catenin and leads to preferential upregulation of negative regulators in response to Wnt3A. (Created with Biorender.com.)

We report in this study that loss of function in *BRCA1* or *BRCA2* results in distinct responses to Wnt signaling in ovarian cancer cells. Although cells with WT and functional *BRCA1*/*BRCA2* activate the canonical Wnt/β-catenin–dependent signaling pathway upon Wnt3A treatment, cancer cells that lose *BRCA1* function activate the noncanonical β-catenin–independent pathway instead. Remarkably, the loss of *BRCA2* function renders ovarian cancer cells with a more stable β-catenin, and these cells respond to Wnt treatment by preferential upregulation of the negative regulator Axin2 (Fig. 7).

Our results show that Wnt3A upregulates canonical β-catenin targets in ovarian cancer cells with functional *BRCA1* and *BRCA2*, particularly the full-length WT Tcf1/7, c-Myc, and cyclin D1. Tcf1/7 is a transcription factor required for β-catenin–dependent gene transcription. The β-catenin protein contains domains with potent transcription activation function but itself lacks DNA-binding capability. Full-length Tcf1/7, with its β-catenin–binding and DNA-binding domains, directs β-catenin to specific DNA sequences to initiate transcription (46). cyclin D1, like Tcf1/7, is also a key target of the canonical Wnt/β-catenin pathway (47). Cyclin D1 is a key regulator of cell-cycle progression by promoting G_1_–S transition and therefore provides a proliferative advantage to cancer cells (48). Myc, another classical Wnt/β-catenin gene target, regulates the cell-cycle similar to cyclin D1 by inducing genes that can promote the progression to both the G_1_ and S phases of the cell cycle (49). In addition, Myc has a significant impact on cellular metabolism and promotes glycolysis, oxidative phosphorylation and a myriad of metabolic pathways required to assure cancer cells of a continued supply of energy and anabolic building blocks to support proliferation, survival, and metastasis (50). It is therefore not surprising that the activation of the canonical Wnt/β-catenin pathway confers several advantages to the growing tumor. Given that we did not observe upregulation of Tcf1/7, Myc, and cyclin D1 in *BRCA2-*null ovarian cancer cells, the potential absence of these prosurvival mechanisms may very well contribute to the improved survival observed in patients with *BRCA2* ovarian cancer (15).

The activation of canonical Wnt signaling in ovarian cancer cells with functional *BRCA1* and *BRCA2* can also directly promote resistance to PARP inhibitors. The use of PARP inhibitors, particularly in *BRCA1*- and *BRCA2*-mutant ovarian cancers, has revolutionized the management of these patients and has been one of the most successful achievements in the last decade in the field. Recently, Yamamoto and colleagues showed that olaparib-resistant ovarian cancer cells upregulate Wnt3A and that forced activation of the canonical Wnt/β-catenin pathway resulting in resistance to both olaparib and rucaparib (28). If PARP inhibitors can promote canonical Wnt/β-catenin signaling by increasing the levels of the Wnt3A ligand, then the diminished responsiveness to canonical Wnt signaling in *BRCA2-*null ovarian cancer cells may also possibly contribute to the improved survival observed in *BRCA2*-mutant patients. Indeed, Wnt signaling inhibition has been shown to confer induced synthetic lethality to PARP inhibitors (51).

In contrast to the activation of canonical Wnt signaling in ovarian cancer cells with functional *BRCA1* and *BRCA2*, our results show that loss of *BRCA1* leads to the activation of the noncanonical Wnt signaling pathway in response to Wnt3A. Typically, and for simplicity, Wnt3A is associated with canonical Wnt signaling, whereas Wnt5A is associated with noncanonical Wnt signaling. Nevertheless, Wnt3A has been shown to activate noncanonical signaling (52). Although not fully characterized, it has been suggested that Wnt signaling is not linear and can activate a vast network at any given time albeit with varying potency. These findings further highlight the complexity of this signaling pathway. Interestingly, in our model, we observed that Wnt3A preferentially activates the noncanonical Wnt signaling pathway in *BRCA1-*null ovarian cancer cells. Particularly, we did not observe upregulation of Tcf1/7, cyclin D1, or Myc but instead saw upregulation in F-actin in response to Wnt3A. F-actin polymers are formed from globular actin monomers (53) and localized in stress fibers and pseudopodia. F-actin is in constant state of flux with globular actin monomers being added from one end and removed from the other (54). This process is important in cancer cell migration and invasion and thus promotes metastasis formation (54). Thus, both canonical and noncanonical Wnt signaling contributes to tumor progression although through distinct processes.

Neither canonical nor noncanonical Wnt signaling was observed in *BRCA2-*null ovarian cancer cell in response to Wnt3A. There were, however, several interesting observations in *BRCA2-*null cells. First, basal levels of nuclear β-catenin were highest in these cells. Second, only Axin2 was upregulated in these cells in response to Wnt3A. Axin2 is an integral component of the β-catenin destruction complex, and its expression is regarded as a rate-limiting step for the function of this complex (37). Overexpression of Axin2 has been shown to promote β-catenin degradation even in adenomatous polyposis coli–mutant cells (55, 56). It is therefore easy to speculate that the upregulation of Axin2 upon Wnt3A treatment may be a negative feedback response from the already high levels of basal β-catenin. Similarly, the observed upregulation of Wnt signaling inhibitors such as Dkk1 and Notum in patients with *BRCA2*mt ovarian cancer may also very well be a negative feedback response. A third observation in *BRCA2-*null ovarian cancer cells is that basal Tcf1/7 is highest in these cells. More importantly, Western blot analysis showed faster migrating bands detected by the Tcf1/7 antibody. Truncated Tcf1/7 “dominant negatives,” which are transcribed from alternate promoter sites and lack the β-catenin–binding domain, have been previously described (39). These proteins cannot bind β-catenin and tend to remain bound to transcriptional repressors on the DNA and act as inhibitors of β-catenin–mediated transcription (38, 46). Alternatively, these Tcf1/7 variants can further provide specificity to gene targets downstream of Wnt signaling. The presence of these truncated Tcf1/7 proteins and their effect on the responsiveness of *BRCA2-*null cells to Wnt3A are currently under investigation. Nonetheless, it can also be considered that the presence of these truncated Tcf1/7 proteins may be the mechanism preventing the transcription of full-length Tcf1/7, Myc, and cyclin D1 and the preferential transcription of Axin2 instead in the *BRCA2-*null ovarian cancer cells.

The demonstration that GSK3β is inactive in *BRCA2-*null cells suggests multiple possibilities given that GSK3β has been shown to have at least 100 substrates and about 500 predicted substrates (43). In addition to impacting the Wnt signaling pathway, GSK3β also feeds into metabolic processes and energy sensing. It is possible that the observed effect on the Wnt signaling pathway may be brought about by these perturbations on GSK3β. How loss of *BRCA2* alters GSK3β is currently under investigation in our laboratory.

In this study, we utilized ID8 mouse ovarian cancer cell lines to validate findings from transcriptomic data obtained from patients with HGSOC. These cells were derived from *in vitro* spontaneously transformed mouse ovarian surface epithelial cells and are to date the most utilized transplantable model of ovarian cancer. Given that the majority of HGSOCs possess p53 loss-of-function mutations, efforts have been made to delete p53 from the ID8 parental cell line to better represent this genetic occurrence in patients (23, 24). The deletion of *BRCA1* or *BRCA2* was made in the background of p53 deletion and provided a platform to compare response to Wnt3A. Given these limitations, it is noteworthy that the observed transcriptomic differences in the Wnt pathway in patients were recapitulated in the ID8 cells.

In summary, we show in this study that loss of function in *BRCA1* and *BRCA2* lead to differential response to Wnt signaling. The activation of canonical Wnt/β-catenin signaling in ovarian cancer cells with functional *BRCA1* and *BRCA2* can confer protumor advantages such as proliferation, survival, and mitochondrial efficiency. Similarly, the activation of the noncanonical Wnt/β-catenin signaling in *BRCA1-*null ovarian cancer cells can confer proliferative and metastatic advantages. In contrast, the unique response to Wnt signaling in *BRCA2-*null ovarian cancer cells can potentially enhance responsiveness to treatment and improve survival. Our findings open venues for the translation of these molecular observations into modalities that can impact patient survival.

## Supplementary Material

Supplementary Table S1 Primers used for qPCR analysis

Figure S1 Chord diagrams of top 10 differentially regulated Pathways and involved DEGs in BRCA2mt vs BRCA1mt

Figure S2 Chord diagrams of top 10 differentially regulated Pathways and involved DEGs in BRCA2mt vs HRwt

Figure S3 Chord diagrams of top 10 differentially regulated Pathways and involved DEGs in BRCA1mt vs HRwt

Figure S4 (A) qPCR analysis of Wnt inhibitors Dkk1, Notum, and Axin2 in ID8 mouse ovarian cancer cells. Data are presented as mean +/- SEM (n=3). Ordinary one-way ANOVA with Dunnett’s multiple comparison test was used to calculate statistical significance; (B) Western blot analysis validates upregulation of Axin2 upon loss of BRCA2.

Figure S5 (A) DEGs in BRCA2null vs BRCA1null 4T1 mouse breast cancer cell line reported as log fold-change (logFC); red bars are upregulated and blue bars are downregulated; (B) Perturbation analysis of Wnt signaling pathway comparing BRCA2null vs BRCA1null 4T1 mouse breast cancer cell line; red genes are predicted to be upregulated and blue genes are predicted to be downregulated.

Figure S6 Nuclear and cytoplasmic green fluorescence were measured in all cells from cultures represented in Figure 3C using image J. Nuclear (left panel) and cytoplasmic (right panel) quantifications are reported separately for each cell line. Statistical significance between control and treated cultures was not achieved.

Figure S7 qPCR analysis for Wnt3A and Ctnnb1 in ID8 mouse ovarian cancer cells. Data are presented as mean +/- SEM (n=3). Ordinary one-way ANOVA was used to calculate statistical significance.
