# Supplementary material for: BRCA Status Dictates Wnt Responsiveness in Epithelial Ovarian Cancer
Source: Cancer Res Commun. 2024 Aug 13;4(8):2075–88. doi: 10.1158/2767-9764.CRC-24-0111 (PMC11320024; doi:10.1158/2767-9764.CRC-24-0111)
Supplement: Supplementary Table S1 — Primers used for qPCR analysis [file crc-24-0111_supplementary_table_s1_suppst1.pdf]

Supplementary Table 1

Primers used for qPCR analysis

Gene	Forward (5'-3')	Reverse (5'-3')
β-catenin	GTTCGCCTTCATTATGGACTGCC	ATAGCACCCTGTTCCCGCAAAG
Axin2	CCACACCCTTCTCCAATCC	TGCCAGTTTCTTTGGCTCTT
Dkk1	GACCATTGACAACTACCAGCCG	TACTCATCAGTGCCGCACTCCT
Notum	CTGCGTGGTACACTCAAGGA	CCGTCCAATAGCTCCGTATG
PPIA	AGCACTGGGGAGAAAGGATT	AGCCACTCAGTCTTGGCAGT
Wnt3A	CACCACCGTCAGCAACAGCC	AGGAGCGTGTCACTGCGAAAG
