# Supplementary material for: BRCA Status Dictates Wnt Responsiveness in Epithelial Ovarian Cancer
Source: Cancer Res Commun. 2024 Aug 13;4(8):2075–88. doi: 10.1158/2767-9764.CRC-24-0111 (PMC11320024; doi:10.1158/2767-9764.CRC-24-0111)

Supplementary Figure 3

Chord diagrams of top 10 differentially regulated Pathways and involved DEGs in BRCA1mt vs HRwt

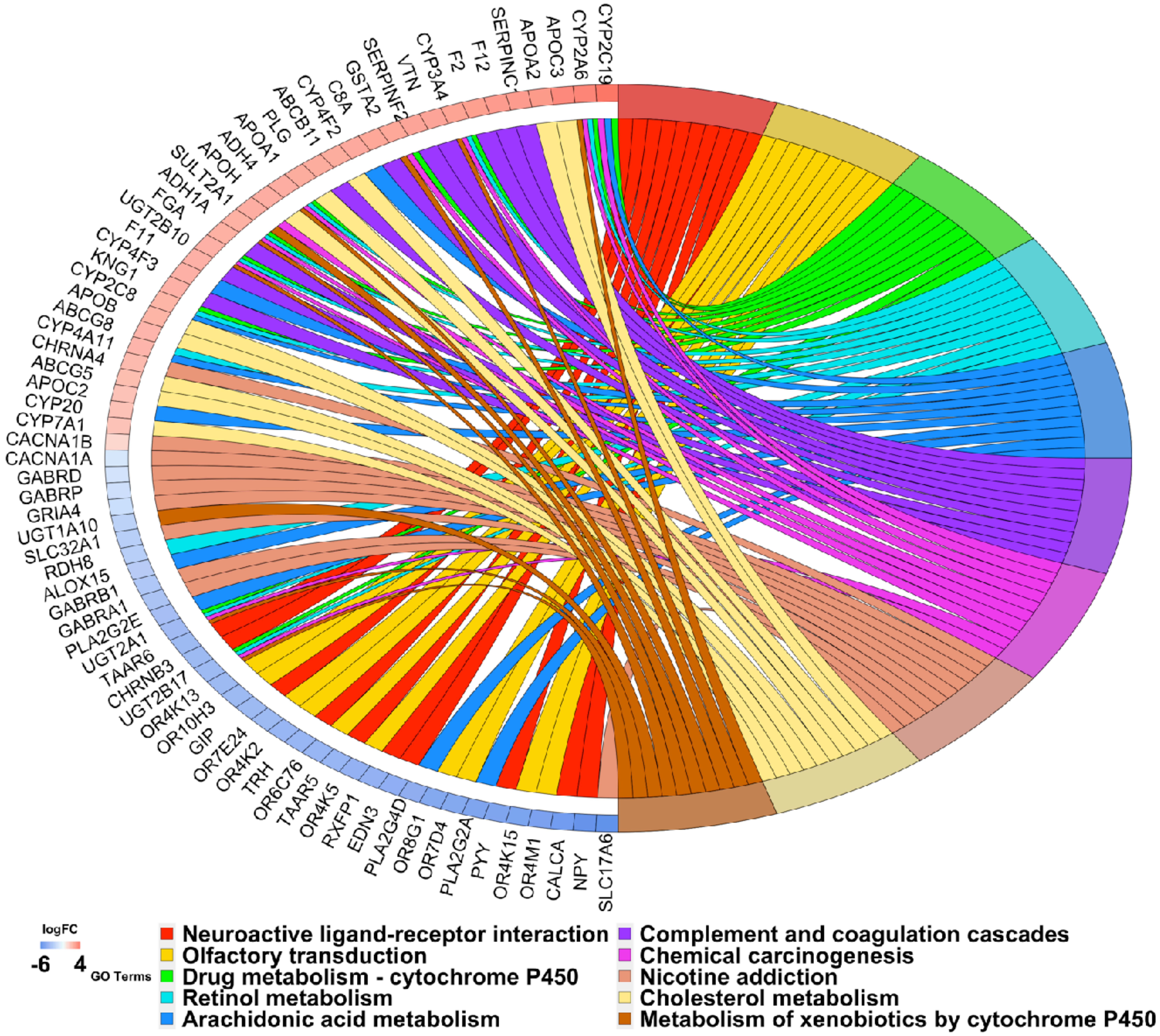

Supplement: Figure S3 — Chord diagrams of top 10 differentially regulated Pathways and involved DEGs in BRCA1mt vs HRwt [file crc-24-0111_figure_s3_suppsf3.pdf]
