# Supplementary material for: BRCA Status Dictates Wnt Responsiveness in Epithelial Ovarian Cancer
Source: Cancer Res Commun. 2024 Aug 13;4(8):2075–88. doi: 10.1158/2767-9764.CRC-24-0111 (PMC11320024; doi:10.1158/2767-9764.CRC-24-0111)

Supplementary Figure 4

(A) qPCR analysis of Wnt inhibitors Dkk1, Notum, and Axin2 in ID8 mouse ovarian cancer cells. Data are presented as mean +/- SEM (n=3). Ordinary one-way ANOVA with Dunnett's multiple comparison test was used to calculate statistical significance; **(B)** Western blot analysis validates upregulation of Axin2 upon loss of BRCA2.

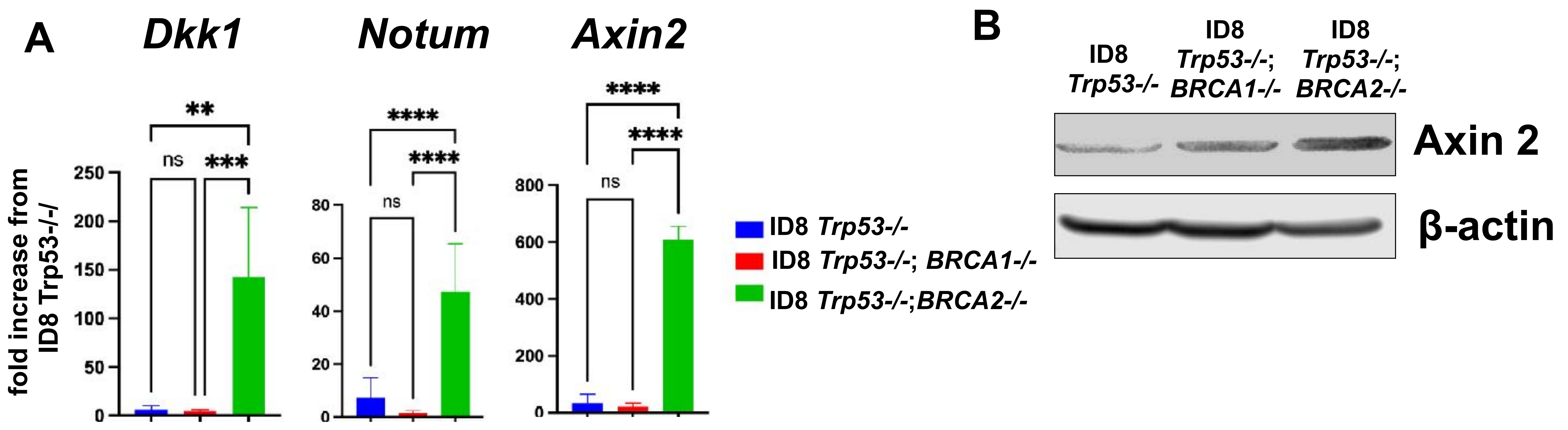

Supplement: Figure S4 — (A) qPCR analysis of Wnt inhibitors Dkk1, Notum, and Axin2 in ID8 mouse ovarian cancer cells. Data are presented as mean +/- SEM (n=3). Ordinary one-way ANOVA with Dunnett’s multiple comparison test was used to calculate statistical significance; (B) Western blot analysis validates upregulation of Axin2 upon loss of BRCA2. [file crc-24-0111_figure_s4_suppsf4.pdf]
