# Supplementary material for: BRCA Status Dictates Wnt Responsiveness in Epithelial Ovarian Cancer
Source: Cancer Res Commun. 2024 Aug 13;4(8):2075–88. doi: 10.1158/2767-9764.CRC-24-0111 (PMC11320024; doi:10.1158/2767-9764.CRC-24-0111)

Supplementary Figure 5

(A) DEGs in BRCA2null vs BRCA1null 4T1 mouse breast cancer cell line reported as log fold-change (logFC); red bars are upregulated and blue bars are downregulated; **(B)** Perturbation analysis of Wnt signaling pathway comparing BRCA2null vs BRCA1null 4T1 mouse breast cancer cell line; red genes are predicted to be upregulated and blue genes are predicted to be downregulated.

A

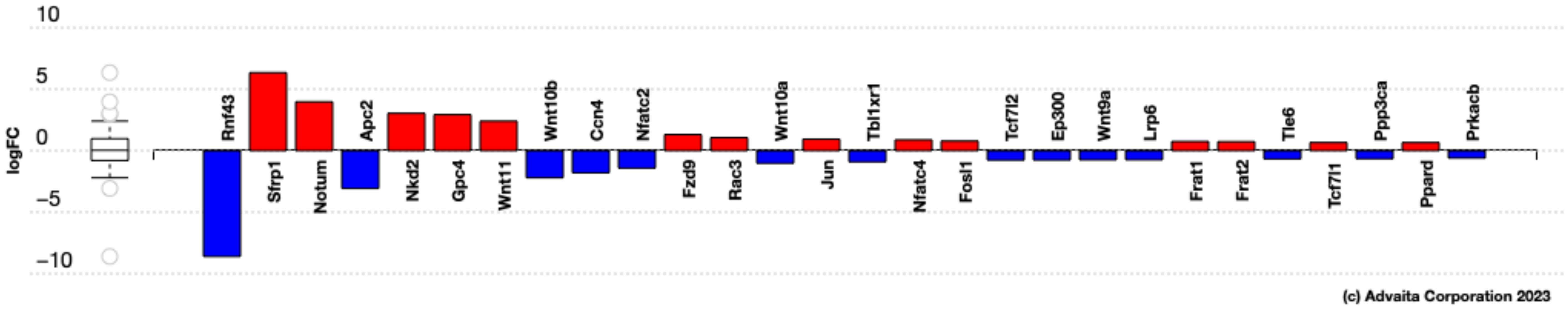

B

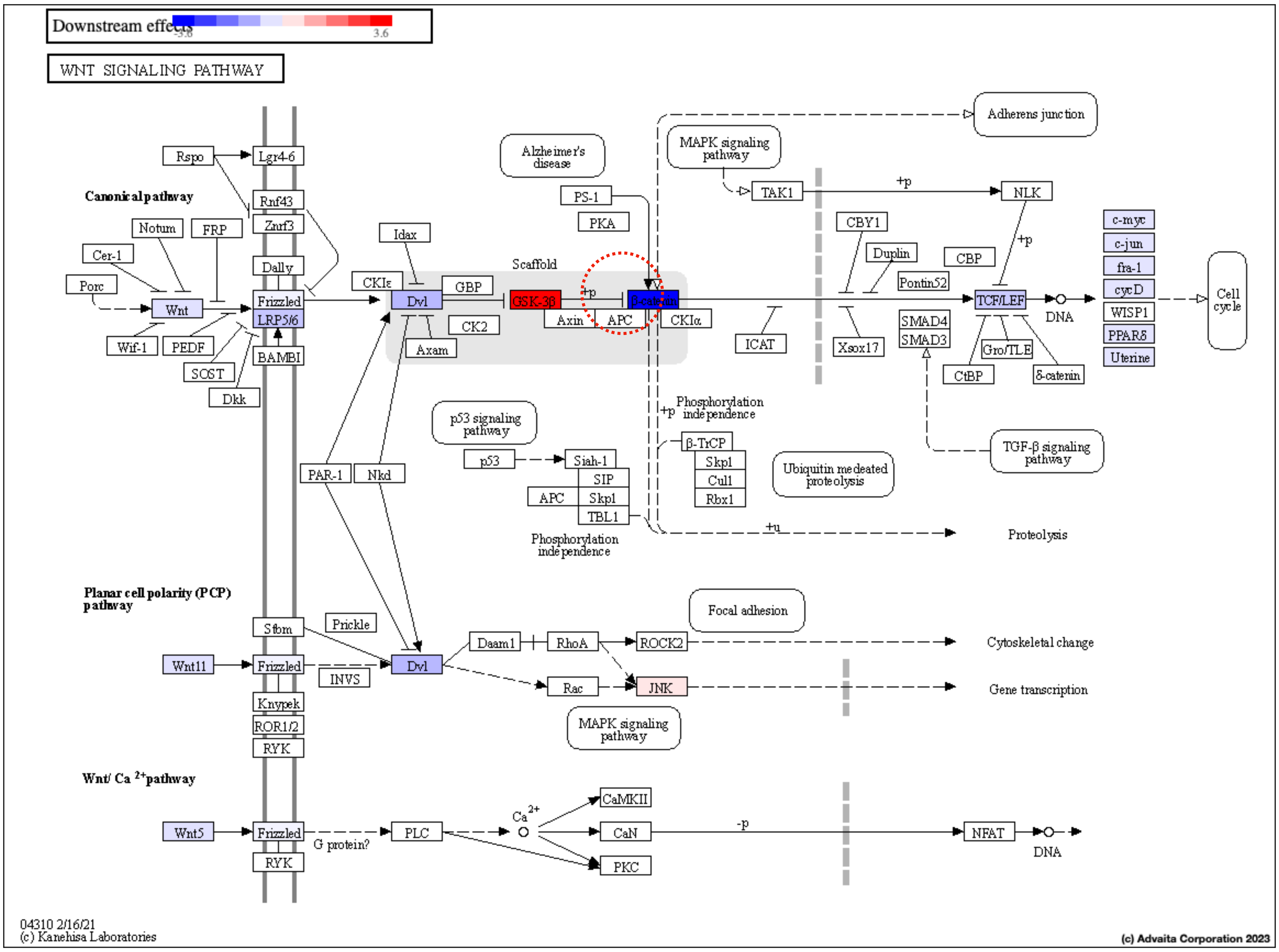

Supplement: Figure S5 — (A) DEGs in BRCA2null vs BRCA1null 4T1 mouse breast cancer cell line reported as log fold-change (logFC); red bars are upregulated and blue bars are downregulated; (B) Perturbation analysis of Wnt signaling pathway comparing BRCA2null vs BRCA1null 4T1 mouse breast cancer cell line; red genes are predicted to be upregulated and blue genes are predicted to be downregulated. [file crc-24-0111_figure_s5_suppsf5.pdf]
