# Supplementary material for: BRCA Status Dictates Wnt Responsiveness in Epithelial Ovarian Cancer
Source: Cancer Res Commun. 2024 Aug 13;4(8):2075–88. doi: 10.1158/2767-9764.CRC-24-0111 (PMC11320024; doi:10.1158/2767-9764.CRC-24-0111)

# Supplementary Figure 6

Quantification of green fluorescence in Figure 3C showing a bigger increase in nuclea green staining (corresponding to β-catenin) in ID8$^{Trp53-/-}$ compared to ID8$^{Trp53-/-;Brca1-/-}$ ar ID8$^{Trp53-/-;Brca2-/-}$cells.

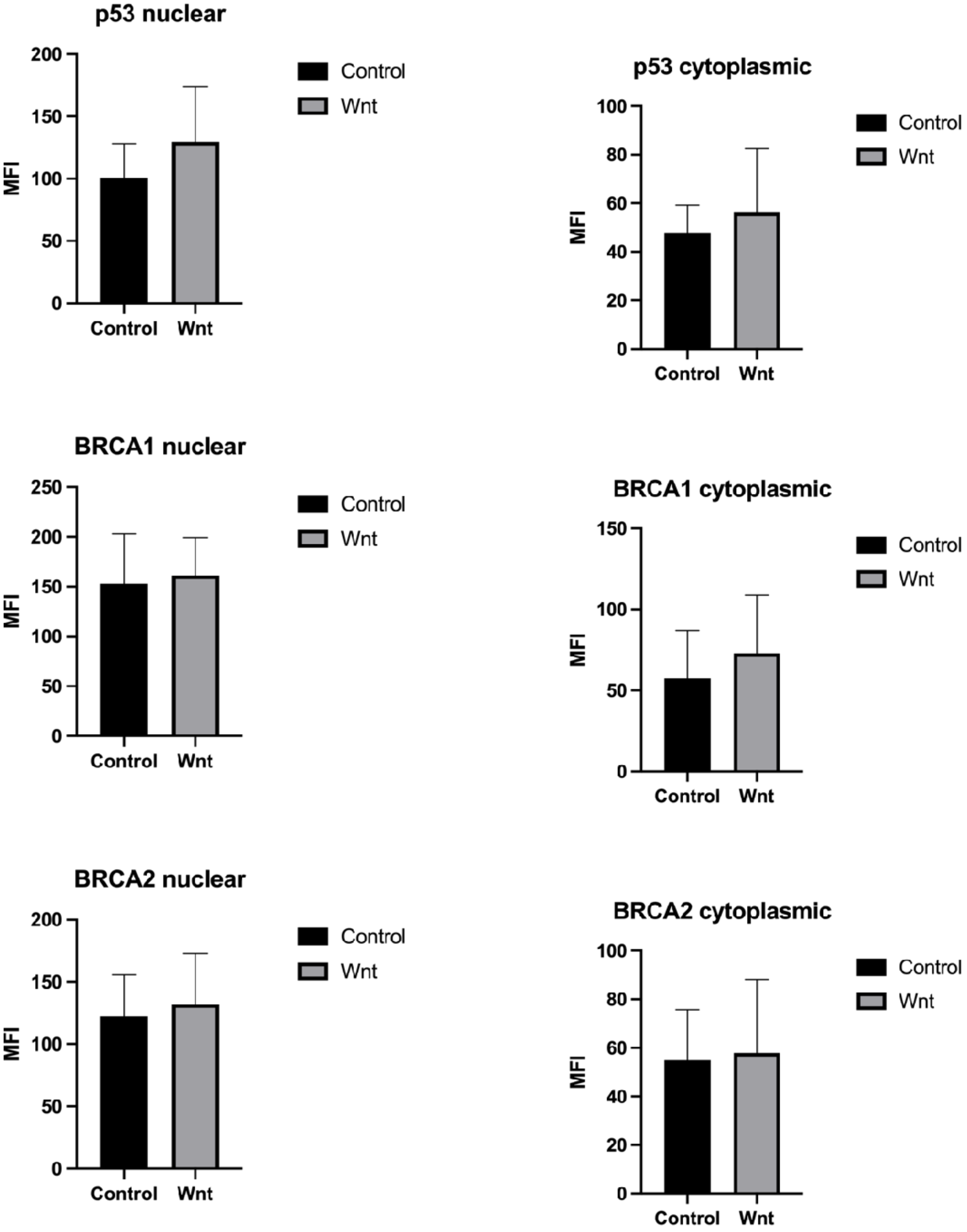

Supplement: Figure S6 — Nuclear and cytoplasmic green fluorescence were measured in all cells from cultures represented in Figure 3C using image J. Nuclear (left panel) and cytoplasmic (right panel) quantifications are reported separately for each cell line. Statistical significance between control and treated cultures was not achieved. [file crc-24-0111_figure_s6_suppsf6.pdf]
