# Supplementary material for: BRCA Status Dictates Wnt Responsiveness in Epithelial Ovarian Cancer
Source: Cancer Res Commun. 2024 Aug 13;4(8):2075–88. doi: 10.1158/2767-9764.CRC-24-0111 (PMC11320024; doi:10.1158/2767-9764.CRC-24-0111)

Supplementary Figure 7

qPCR analysis for *Wnt3A* and *Ctnnb1* in ID8 mouse ovarian cancer cells. Data are presented as mean +/- SEM (n=3). Ordinary one-way ANOVA was used to calculate statistical significance.

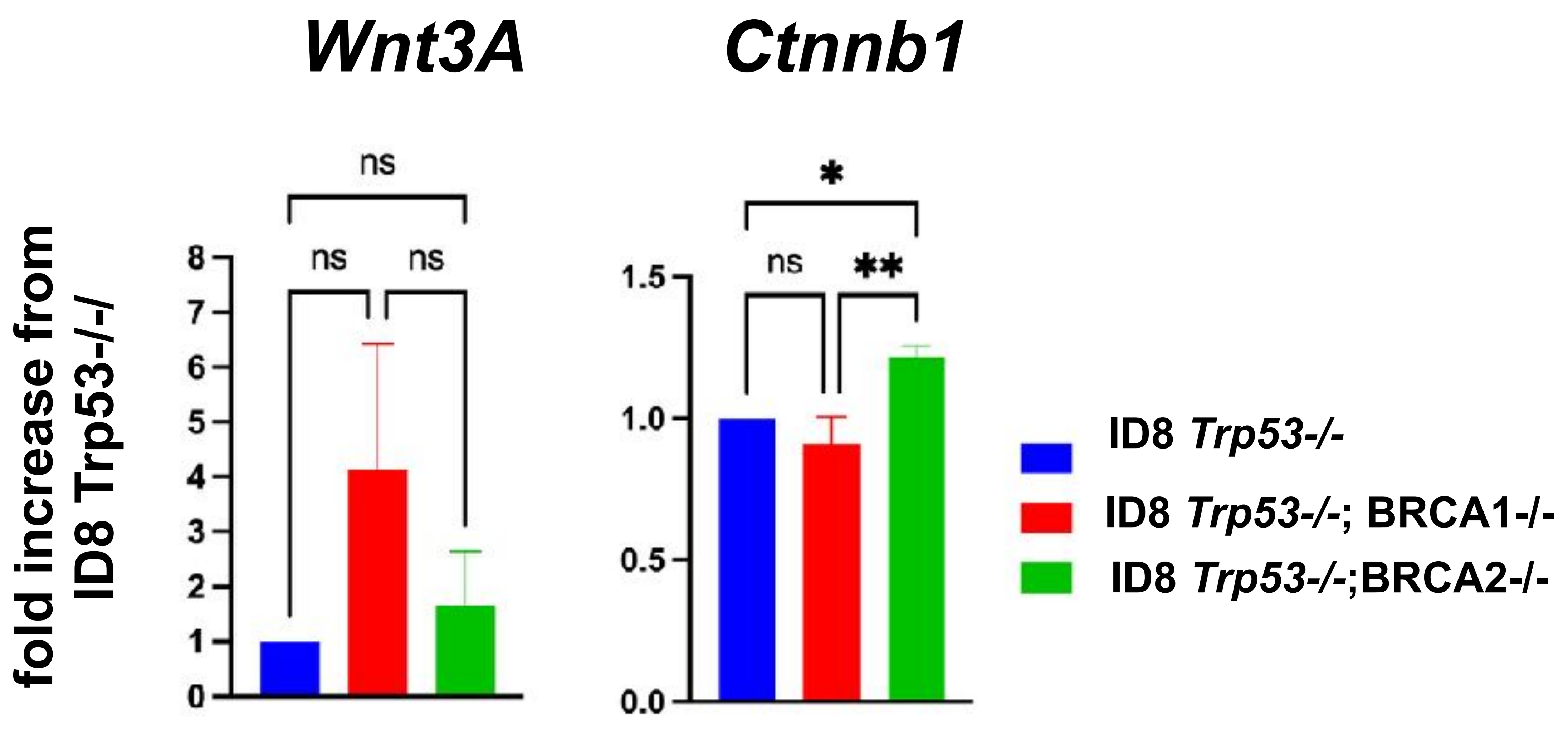

Supplement: Figure S7 — qPCR analysis for Wnt3A and Ctnnb1 in ID8 mouse ovarian cancer cells. Data are presented as mean +/- SEM (n=3). Ordinary one-way ANOVA was used to calculate statistical significance. [file crc-24-0111_figure_s7_suppsf7.pdf]
